# Evaluation of the Proximal Tibia as a Donor Site of Cancellous Bone for Intraoral Grafting Procedures—A Retrospective Study

**DOI:** 10.3390/jcm11061493

**Published:** 2022-03-09

**Authors:** Piotr Malara, Nadine von Krockow, Iwona Niedzielska, Beata Malara

**Affiliations:** 1 Department of Maxillofacial Surgery for Children, Chorzow Hospital for Paediatrics and Oncology, 41-500 Chorzow, Poland; 2 Postgraduate Educational Centre of Dentistry DENTARIS, School of Medicine, Katowice Business School, 40-659 Katowice, Poland; 3 Department of Postgraduate Education, J. W. Goethe University Frankfurt am Main, 60596 Frankfurt am Main, Germany; krockow@med.uni-frankfurt.de; 4 Department of Cranio-Maxillofacial and Oral Surgery, Medical University od Silesia, 40-027 Katowice, Poland; niedzielska.konsultant@wp.pl; 5 Department of Facial Aesthetics and Cosmetology, School of Medicine, Katowice Business School, 3 Harcerzy Wrzesnia Str., 40-659 Katowice, Poland; beata.malara@gwsh.pl

**Keywords:** bone grafting, alveolar bone regeneration, jawbone reconstruction, tibia, odontogenic cyst, mandible

## Abstract

Background: Autogenous bone grafts remain the “gold standard” in maxillofacial reconstructive procedures. The objective of this study was to evaluate the proximal tibia as a donor site of cancellous bone for bone grafting procedures of the mandible on the basis of intraoperative parameters and clinical observations. Methods: The study was based on a medical record search of 40 patients who underwent surgical procedures because of benign pathological lesions of the jaws resulting in 3-wall bone defects of the mandible and qualified for surgical removal of the lesion with simultaneous bone grafting of the defect with autogenous cancellous bone harvested from the proximal tibia. Results: The use of the proximal tibia for bone grafting procedures enables large amounts of cancellous bone (15.09 cc in average) to be obtained. The procedure is characterized by a low risk of early and late complications, which include excessive bleeding, wound infection, lengthy healing time, scars, a loss of sensation around the scars, aching, a dip in bone, swelling and tenderness. Conclusions: The ability to obtain large amounts of cancellous bone and a low risk of intra- and postoperative complications make the proximal tibia an attractive donor site for the bone grafting procedures in maxillofacial surgery.

## 1. Introduction

One of the current problems of up-to-date implant dentistry is alveolar bone deficit in a vertical or transversal dimension. Remodeling of the alveolar bone is a natural consequence of tooth loss by extraction or injury. It begins in the first few minutes after tooth extraction or avulsion and leads inevitably to some degree of atrophy of the alveolar process [1,2]. The bone deficit may be even larger when pathological conditions, such as the presence of impacted teeth, cysts, bone inflammation of various origins, benign and malignant tumors in the bone, cleft palate, as well as other congenital craniofacial malformations are present [3,4].

In cases of bone deficit, contemporary oral and maxillofacial surgery has many methods of treatment aimed at local alveolar bone regeneration. For this purpose, various techniques are used, including guided tissue regeneration [5], bone grafts [6], and distraction osteogenesis [7]. In cases requiring a surgical reconstruction of alveolar defects of a significant size, despite the considerable inconvenience of having to operate at the donor site, autogenous bone grafts remain the “gold standard” [8]. A prerequisite for the clinical success of this method is to provide the graft at the recipient site with an adequate supply of oxygen and nutrients [9].

Bone grafts are divided into several types depending on their structure, immunological response, as well as anatomical and embryologic origin. Depending on the histological structure, the grafts are divided into cortical (skull, chin, the mandibular body, and ramus), cancellous (tibia), and mixed cortico-cancellous bone blocks (iliac crest) [8]. Depending on the embryological origin, the grafts are divided into membranous—derived from mesenchymal cells (all bone grafts from the facial skeleton)—and enchondral—ectomesenchymal cell-derived (tibia, iliac crest) [10]. Up till now, the autogenous bone has been the only source of osteogenic cells that can be used to reconstruct bone defects in oral and maxillofacial surgery. Constantly expanding knowledge of the biology and behavior of bone grafts allows for continuous improvement of clinical protocols, shaping, storage, and fixation at the recipient site [11]. Although recombinant human bone morphogenetic proteins (rh-BMP) appear a promising alternative to autogenous bone grafts, further dose and carrier optimization needs to be investigated to expand their efficacy, use and clinical application in oral and maxillofacial surgery [12,13]. A greater understanding of underlying cellular and molecular mechanisms is required to use rh-BMPs for reconstruction and regeneration of mandibular bone [14].

Taking into consideration the biology of bone graft healing, it must be noted that in the human body there are two different types of bone tissue—cortical and cancellous bone. In oral and maxillofacial surgery, the most widely used donor site for autogenous bone grafts is the iliac crest [15,16]. At this stage, the iliac crest remains the gold standard in large alveolar bone defects [17]. This site enables the harvesting of large volumes of cortical bone, even above 100 cc, and smaller amounts of cancellous bone from 4.98 to 33.57 cc (average of 17.63 cc) [18]. However, the surgical procedure is highly invasive and encounters a significant risk of post- and intra-operative complications [19,20,21,22,23]. The tibia is rarely chosen as a donor site for reconstructive procedures in oral and maxillofacial surgery [8]. In the literature, it is emphasized that this site allows the procurement of large volumes of cancellous bone ranging from 10 to 42 cc [24,25]. It is also noted that the anatomy of the proximal tibia practically excludes the possibility of intra-operative complications. There is little information available about serious complications of the surgical procedure [26,27,28,29,30,31].

Choosing a donor site for reconstructive procedures in surgery, the appearance of the postoperative scar must be taken into account. Scars are the undesirable consequences of any surgical intervention and can significantly decrease the quality of life of the patient [32,33]. Therefore, it is important to provide a patient with information about a possible appearance of the scar at the donor site when obtaining the informed consent of the patient for surgery. Preference should be given to donor sites that are inconspicuous and allow for short skin incisions.

Thus, it seems important to carry out a critical evaluation of selected intra- and post-operative parameters of cancellous bone harvesting procedures from the proximal tibia and to compare them with the available information on surgical bone harvesting from other extraoral sites.

The objective of the study was to evaluate the proximal tibia as a donor site of cancellous bone for bone grafting procedures of the mandible on the basis of intraoperative parameters and the clinical outcome.

## 2. Materials and Methods

### 2.1. Study Population

The data includes medical records of 40 patients who underwent surgical procedures between June 2016 and March 2021 because of odontogenic cysts of the mandible resulting in 3-wall bone defects. All the surgical procedures were performed by the same specialist in maxillofacial surgery, who in 2016 had 9 years of clinical experience as a consultant in his specialty (P.M.). The patients were qualified for surgical removal of the cyst with simultaneous bone grafting of the defect using autogenous cancellous bone harvested from the proximal tibia. All the patients included in the study were Caucasians. The study group consists of 26 women and 14 men aged 19 to 67 years. All the patients had undergone a minimum 6-month follow-up period since the surgical treatment.

### 2.2. Diagnostics and Pre-Surgical Procedures

Clinical examination was carried out and orthopantomograms (OPG) were taken for all the patients. The size of the lesion, its relationship to surrounding structures, the borders of the lesion, appearance of the surrounding bone, and the possible presence of a causal tooth were observed on the OPG. Patients qualified for removal of a lesion with simultaneous cancellous bone grafting from the tibia when the largest diameter of the lesion was above 4 cm. If the appearance on the OPG proved the benign character of the lesion and the clinical examination did not reveal any disruption of the surrounding cortical bone, no further radiographs were taken. If the borders of the lesion were blurry, the lesion had irregular or polycyclic shape, or there was no causal tooth that could prove an odontogenic character of the lesion, computed tomography (CT) scans and biopsies from the lesion were taken. Having proven a benign character of the lesion, the patients qualified for the removal of the lesion with simultaneous cancellous bone grafting from the tibia.

Posteroanterior (PA) and lateral views of the knee joints were taken in all the patients qualified for the bone harvesting procedures from the proximal tibia. In the process of selection of a limb to harvest cancellous bone, the data from the case history (no previous fractures, no previous bone pathologies) were taken into account. Patients who reported current or past difficulties in walking, undiagnosed pain in the area of the knee joints and previous surgery in the lower limbs did not qualify for cancellous bone harvesting from the proximal tibia. However, at this stage, no additional medical examination was performed to identify the presence or absence of pre-existing conditions, mainly alignment abnormalities or arthritis. The current knowledge on the collection of cancellous bone from the proximal tibia does not allow for prediction of the bone volume that can be harvested during surgery based on preoperative radiological examinations only. The amount of bone that can be harvested must be assessed intraoperatively. Therefore, the patients where it was possible to collect cancellous bone from both tibiae, were asked to consent to the harvesting of the bone from both limbs, just in case the bone volume taken from only one limb would turn out to be insufficient for grafting the whole mandibular defect. However, if both limbs could constitute donor sites, the patient’s first preference was taken into consideration.

### 2.3. Surgical Protocol

**Figure 1:**
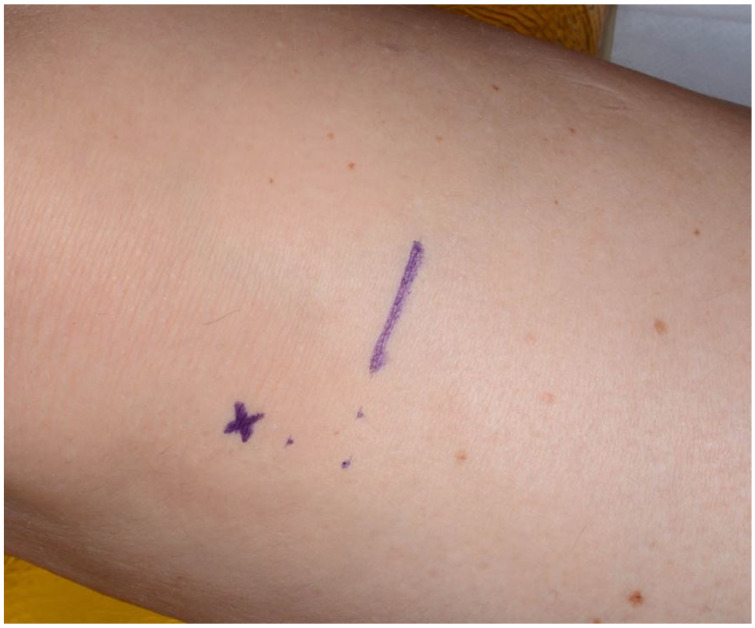
Incision line for the medial approach to the proximal tibia. Legend: “x” indicates the middle of the tibial tuberosity; the dots are marked 1 cm apart one from another.

All the operative procedures were carried out under general anesthesia with endotracheal intubation. The skin around the knee joint and the proximal tibia was washed twice with a surgical disinfectant and the operative field was wrapped with drapes (Figure 1).

**Figure 2:**
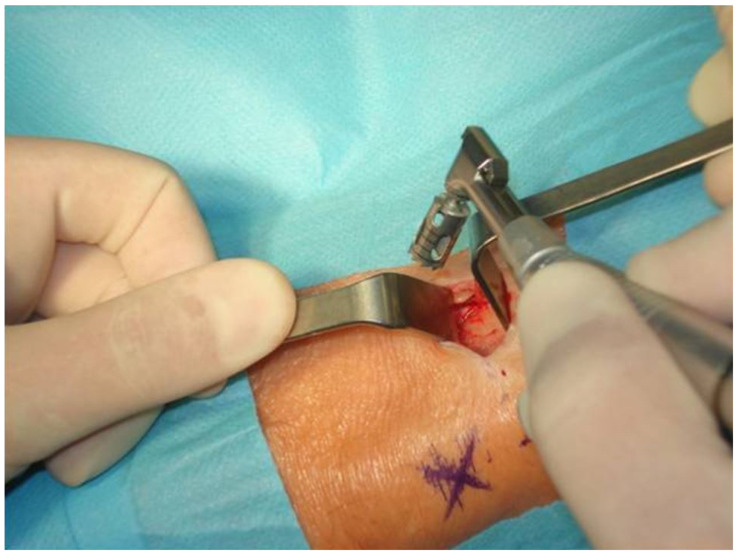
An opening in the cortical layer is made with a trephine bur 8 mm in diameter.

The site of the incision was infiltrated with 2 cc of 2% lidocaine with noradrenaline (1:100,000) down to the periosteum. A line of surgical incision with a length of 20–30 mm was drawn 2 cm below and 2 cm to the middle of the tibial tuberosity. A through-and-through incision was made with a blade No. 10. The periosteum was reflected with a raspatorium to ensure easy access to the bone. The soft tissues were held with two Langenbeck hooks. An opening in the cortical layer was made with a trephine bur 8 mm in diameter mounted on a contral-angle surgical handpiece with a rotary speed of 120 revolutions per minute and copious irrigation with sterile 0.9% saline solution (Figure 2).

**Figure 3:**
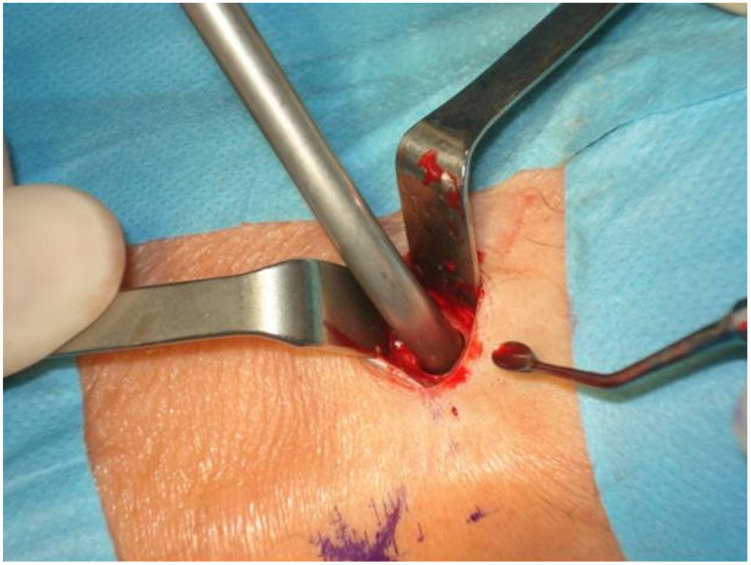
A bone collector attached to the vacuum suction is used to collect the bone.

Having cut out the window in the cortical bone with a trephine, no attempts of direct harvesting of the cancellous bone were made. Instead, the cancellous bone was separated from the cortical layer with straight and curved curettes. A commercially available bone collector (Bone Vac produced by Stryker, Portage, MI, USA) attached to the vacuum suction was used to collect the bone (Figure 3). During the cancellous bone collection, copious irrigation with 0.9% sterile saline solution was performed. The procedure was carried out until there was no more cancellous bone available or retrievable through the bony window. The cortical bone taken out during the window opening was particulated in a bone mill (SD-Bone Mill produced by Surgident Co. Ltd., Dong-gu, South Korea), mixed with the cancellous bone retrieved from the proximal tibia, and used for the grafting procedure.

The skin incision was closed in three layers. The periosteum was closed with absorbable 3-0 suture, the subcutaneous tissue with absorbable 4-0 stitches, and the skin was closed with monofilament intradermal 5-0 suture. The wound was protected with a sterile dressing.

### 2.4. Postoperative Care

Just after the operation the shaft from the foot up to the knee was wrapped with an elastic bandage to prevent the formation of a hematoma. A few hours after the surgery the patients were encouraged to walk. However, the patients were instructed to avoid an excessive load on the limb (jumping, running, climbing on a ladder) within 3 months after surgery. A control X-ray of the tibia was taken to verify the correctness of the execution of the hole in the cortical bone and to exclude any fractures or infractions of the tibia. Sutures were removed between 7 and 10 days after the surgery. The hospitalization period depended on the type of operation in the maxillofacial skeleton.

Checkup visits were appointed in the outpatient department after 3 and 6 months. During these visits, particular attention was paid to possible pathologies at the recipient and the donor site, and the appearance of the skin scar at the donor site was assessed. A detailed interview on the postoperative period was carried out.

### 2.5. Evaluated Parameters and Measurements

The research work includes in particular the evaluation and measurements of selected parameters:

(1) Calculation of the Body Mass Index (BMI) of the patients
(2) The length of the skin incision
(3) Surgery time from the skin incision until the end of the skin suturing
(4) Surgery time from the bone opening with a trephine until the beginning of the suturing of the periosteum
(5) A quantity of the harvested cancellous bone
(6) Time from the surgery endpoint until patient mobilization (start of walking)
(7) The number of consecutive days when the patient experienced pain at the donor site requiring the taking of painkillers
(8) The number of consecutive days when the patient s limped
(9) The number of consecutive days when the patient needed a walking aid (a walking stick)
(10) Early complications (excessive and prolonged bleeding, hematoma requiring a surgical intervention, infection, wound dehiscence, wound suppuration)
(11) Late complications (fractures of the tibia, aching, prolonged limping, lengthy healing time, scar, loss of sensation around the scar, swelling, and tenderness)
(12) Possible relationship between the length of the skin incision and the total time of the procedure of the bone harvesting from the proximal tibia
(13) The survival rate of the graft after 6 months assessed on orthopantomograms.

BMI was calculated based on the patient’s height and weight stored in the medical records.

The length of the skin incision was measured with a sterile surgical ruler. The measurements of the time of the operation (the time from the skin incision until the end of the skin suturing, the time from the bone opening with a trephine until the beginning of the suturing of the periosteum) were taken by an assisting nurse. The measurements of the cancellous bone volume were carried out before the bone was placed at the recipient site using a standard sterile Luer syringe with a volume of 30 cc. For measurements, the syringe plunger was removed. The cancellous bone was loosely placed in the syringe without exerting any excessive compression and the readings were performed on the scale on the side of the syringe.

The time from the surgery endpoint until the patient mobilization (start of walking) and the information on early complications (excessive and prolonged bleeding, hematoma requiring a surgical intervention, infection, wound dehiscence, wound suppuration) were achieved with the postoperative nursing care protocol. The number of consecutive days when the patient had experienced pain at the donor site requiring the taking of pain-killers, the number of consecutive days when the patient had limped, the number of consecutive days when the patient needed a walking aid (a walking stick), and the information on late complications (fractures of the tibia, aching, prolonged limping, lengthy healing time, scar, loss of sensation around the scar, swelling, and tenderness) were acquired during the interviews at the check-up appointments. The survival rate of the grafts was assessed on orthopantomograms on the 6-month check-up appointments. If there were no radiological changes in the bone structure at the area and around the graft, the procedure was assumed to be successful.

### 2.6. Statistical Analysis

The normality of the distribution of the analyzed parameters was tested with the Shapiro-Wilk test. The statistical significance of differences between the analyzed parameters was tested with Student’s *t*-test when variables showed a normal distribution and with the U Mann-Whitney test when variables did not show a normal distribution. The difference in incidence of a feature was estimated with a Chi^2^ test. An analysis of the correlation between parameters that have a logical relationship from a clinical point of view was also carried out. The relationship between the length of the skin incision and the duration of the operation was analyzed using Pearson’s correlation coefficient as both the parameters had characteristics of a normal distribution. Statistical significance was analyzed at the *p*-level < 0.05. Statistical analysis software Statistica 10.0 was used. BMI was treated as a continuous variable in order to compute the mean value in the whole study group and with regard to the patient’s gender.

## 3. Results

The mean age of the study population was 38.70 ± 14.23 years (36.62 ± 11.44 for women; 41.50 ± 15.33 for men) BMI of the study population ranged from 17.4 to 33.8 (mean 24.2 ± 8.3 for the whole group; 27.3 ± 8.7 for women; 21.4 ± 6.4 for men). The results of the Shapiro-Wilk test to check the normality of distribution showed that only the results of the length of the skin incision, the surgery time from the skin incision until the end of the skin suturing, and the surgery time from the bone opening until the beginning of the suturing were normally distributed. The other parameters under analysis did not show characteristics of a normal distribution.

Intraoperative parameters analyzed for the whole group of patients are shown in Table 1. The average length of the skin incision performed to gain access to the proximal tibia was 25.21 ± 3.23 mm. The average time from the start of the skin incision until the end of the skin suturing ranged from 37 to 52 min (44.21 ± 3.59 min on average). The time of soft tissue management involving the soft tissue preparation on reaching the surface of the cortical bone of the tibia and then a layered suturing of the soft tissues was 20.47 ± 2.13 min on average. The time needed for making an opening in the cortical bone with a trephine bur and collection of the cancellous bone ranged from 19 to 29 min (23.74 ± 3.01 on average), which allowed for the acquisition of 8 to 21 cc of the cancellous bone (15.09 ± 3.75 on average). This volume of cancellous bone proved to be sufficient for complete filling of the mandibular defects created as a result of surgical enucleation of the mandibular cysts in all the patients treated with this technique. Although the patients were asked to consent to the harvesting of the cancellous bone from both limbs, there was never the necessity to collect the bone from both proximal tibiae, as the amount of bone taken from one side was sufficient to graft the mandibular defect. The analysis of the intraoperative parameters taking into account the patient’s gender revealed a statistically significant difference for the length of the skin incision between the group of women and men (Table 2). There were no statistically significant differences between the other intraoperative parameters. It must be noted that there was no statistically significant difference in the mean BMI between the group of men and the group of women if the parameter was treated as a continuous value. However, when BMI was recorded into categories (below 18.5-underweight; 18.5–24.9 normal weight; 25.0–29.9 overweight; over 30.0-obesity), the mean BMI of women fell into the overweight category and the mean BMI of men indicated the normal weight.

**Figure 4:**
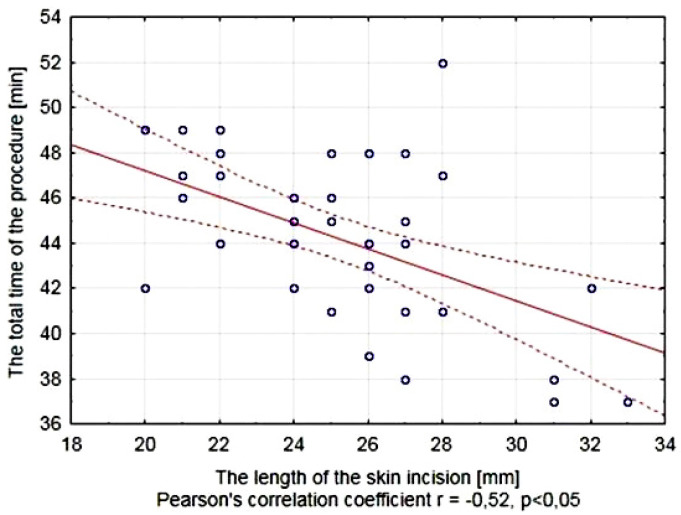
The relationship between the length of the skin incision and the total time of the procedure of bone harvesting from the proximal tibia.

The analysis of the relationship between the length of the skin incision and the total time of surgery showed a significant negative correlation between these parameters, characterized by Pearson’s correlation coefficient r = −0.52. A longer incision allows for better soft tissue retraction and an easier access to the bone surface resulting in significant shortening of the operation time (Figure 4).

The early postoperative period was characterized by the time from surgery endpoint until patient mobilization (patient’s attempts to walk alone) and the number of days with pain at the donor site requiring the taking of painkillers. The data analyzed for the whole group of patients are shown in Table 3. The mean time from surgery endpoint until patient mobilization was 5.40 ± 1.14 h and did not exceed 11 h. The patients experienced pain forcing them to take painkillers for a period of time not exceeding 14 days (8 days on average).

In the analyzed group of patients, the walking difficulties manifested by limping, which occurred in 29 individuals represented 72.5% of the study population (Table 4). On average, symptoms persisted in these patients for about 3 weeks. The remaining 14 patients reported no difficulties in ambulation. The longest lasting limping occurred in a 61-year-old patient who additionally reported pain at the donor site even at a follow-up visit after 6 months. In the group of patients who experienced walking difficulties in the postoperative period, 10 patients (25.0%) needed to use a walking stick for a maximum three weeks (13 days on average).

Early postoperative complications included excessive bleeding from the wound in three patients (two women 61 and 67 years old and one 52-year-old man). In the same 61-year-old woman, an infection of the post-operative wound appeared (Table 5). It should be noted that heavy bleeding occurred only in elderly patients and none of these cases required any surgical revision of the wound. The bleeding disappeared after applying an additional pressure dressing. In the case of infection, the intervention was limited to the removal of two stitches allowing for evacuation of pus. There was no need to drain the wound. No additional antibiotic treatment was necessary in this case.

The patient who had a postoperative wound infection also developed late complications in the form of prolonged wound healing (the only such complication among all analyzed patients) and a non-cosmetic postoperative scar. Late complications included aching at the scar area in two cases, loss of sensation around the scar in one case, a palpable dip in bone in one case and persistent swelling and tenderness at the donor site for a period of more than four months in one case (Table 6).

OPG’s taken 6 months postoperatively showed no pathological changes in the area and around the graft in all the cases in the study, so that survival of the cancellous bone graft was assumed within the limitations of this retrospective study design.

## 4. Discussion

The problem of the choice of a donor site for pre-implantation bone grafting is still current. It is assumed that among numerous criteria the most important are the preferences and the experience of the operator, the quality of the bone in the donor site, the morbidity of the donor site associated with the procedure, and the possibility of early and late complications [34]. According to many authors, the most important criterion should be the volume of the bone available at the donor site suitable for augmentation in the recipient site [23,35]. Because of the current state of knowledge, it is impossible to predict precisely the bone volume that would be possible to collect during the surgery based solely on radiological examination, the technique of cancellous bone harvesting from the tibia was identical in all patients, which made it possible to objectively compare the parameters assessed in our study. The aesthetic result of the procedure is also very important. It is affected by the ability to achieve shortest possible and nearly invisible scars and to hide them in unexposed regions of the body [33].

The length of the skin incision needed for a comfortable access to the bone plays an important role in choosing the donor site. A shorter incision affects the time of soft tissue preparation, reduces the suturing time, decreases the risk of intraoperative and postoperative infection by minimizing the size of the gates for micro-organisms, reduces the risk of major bleeding and damage to the nerve fibers, and ultimately leads to a reduction of the scar size [33]. The authors of many publications agree that to collect the cancellous bone from the proximal tibia by the medial approach it is sufficient to make a 20–25 mm long skin incision [36,37]. In our own group of operated patients, the average length of skin incision was 25.21 ± 3.23 mm. Significantly longer incisions were performed in women (26.10 ± 3.03 mm) than in men (23.36 ± 2.90 mm), which was probably associated with a slightly thicker layer of fat tissue in women in this area. It corresponds to the fact that the mean BMI calculated with regard to the patient’s gender indicated overweight in the group of women and normal weight in the group of men. It should be noted that the extension of the incision line significantly affects the total time of bone harvesting by facilitating the access to the bone surface (Figure 4). This is consistent with the observations of other authors in other types of procedures in orthopedic surgery [15,38].

The duration of surgery is very important for the course of post-surgery tissue healing and the pain experienced by a patient. In the analyzed material, the average bone harvesting time from the proximal tibia was 44.21 ± 3.59 min. The total duration of the procedure consists of the soft tissue surgery time, which was 20.47 ± 2.13 min and the time of operation on hard tissues (23.74 ± 3.01 min). The total bone harvesting time does not differ significantly between the group of women and the group of men. It should be noted that the analyzed group of patients was subjected to surgery under general anesthesia, which was dictated by the extent of the surgery in the oral cavity. The procedure can be successfully carried out in intravenous sedation [29,30,31]. In most research works there is no information regarding the duration of the procedure. Marchena et al. [31] reported the mean duration of the bone harvesting from the proximal tibia to be equal to 58 min. It is a longer time than the time recorded in our own data, but it is probably related to surgery under sedation rather than under general anesthesia.

In our own data, the mean volume of the harvested bone was 15.09 ± 3.75 cc (up to 21 cc) (Table 1). There were no differences in the amount of the bone in relation to the patient’s gender (Table 2). Some authors report on larger volumes of bone harvested, ranging from 10 to 42 cc [24,25]. Vittayakittipong et al. [37] studied the average volume of cancellous bone possible to obtain from this area on cadavers and found that the average volume of the harvested material was 14.58 ± 3.30 cc. In comparative studies using three-dimensional imaging model for the assessment of the amount of cancellous bone in the proximal tibia, even greater volumes of the material were found ranging from 16.26 to 69.56 cc (average of 38.60 cc) [18]. It should be noted that in all the cases analyzed in our study, the amount of cancellous bone harvested was sufficient for the intraoral augmentation procedures. It is not always possible to predict the amount of bone that will be needed for grafting. It is recommended to obtain the patient’s consent for bone harvesting from both tibiae [26,29].

Post-operative pain and discomfort are natural consequences of all surgical procedures. In choosing a donor site for reconstructive procedures, the morbidity must be considered. It is very difficult to assess pain because different researchers use different definitions and methods of evaluation. Assessment is accomplished at various points of time after surgery, and there are various surgical approaches and different protocols of postoperative pain management in different studies [39]. Pain assessment is extremely difficult because patients differ in their capacity for reception and evaluation. Analog numerical scales are most commonly used for this purpose. They are considered to be simple to implement and the results repeatable [34,40]. In our own data pain was assessed on the basis of the number of days in which patients needed to use analgesics (Table 3). The average number of days in which the patients took painkillers was 7.95 ± 2.55 days (at least one, maximum 14 days). SooHoo and Cracchiolo [41] did not report any severe pain in patients after procedures of cancellous bone harvesting from the tibia. Marchena et al. [31] report that in their group of 10 patients, postoperative discomfort was experienced for an average of 9.8 days (from 2 to 39 days), but the authors do not specify whether the discomfort required administration of analgesics. Kirmeier et al. [30] in the analyzed group of 79 patients undergoing cancellous bone harvesting from the proximal tibia found significant pain lasting up to 14 days in 12 patients (15.2%) and over 14 days in four patients (5.1%). Although postoperative pain is evaluated using various methods, in most reports cancellous bone harvesting from the proximal tibia bone causes pain described as moderate [8,42,43].

An early patient mobilization after surgery has many advantages and results in a reduction of complications associated with immobility and eliminates the need for third-person assistance during rehabilitation. In our own research, the patients were encouraged to walk on the first postoperative day and the average time from the end of surgery to the first attempt to walk was 5.40 ± 1.14 h (Table 3).

The consequence of most operative procedures carried out within the lower limbs are gait disturbances, often forcing the patient to use a walking aid in the form of a walking stick or crutches. In the analyzed data the gait disturbances occurred in 29 patients (72.5%) (Table 5). The average duration of these symptoms in the whole group was 20.59 ± 14.76 days (from 2 to 86 days). Ten patients required walking aids within a mean period of 13.30 ± 4.30 days (7 to 21 days). Hughes and Revington [26] found gait disturbances in 31 of 75 patients, within an average period of 17 days (from 2 to 12 days). Marchena et al. [31] report that in the group of 10 patients studied, limping occurred for an average of 9.4 days postoperatively (from 1 to 39 days) and was correlated with patient weight.

Short-term pain following surgery accompanies every surgical intervention. Another problem is long-lasting pain after surgery, which falls into a category of late complications of the surgery. In our data swelling and tenderness of the lower limb occurred in one case (2.5%) (Table 6). This patient worked as a miner in a coal mine. Probably the occurrence of this complication was caused by the work conditions and excessive load on the limb. Another problem is the occurrence of aching in the scar, which is caused by scar tissue interaction with nerve endings. The risk of skin numbness occurs in each case of skin incision. In the analyzed data the sensory disturbances in the scar occurred in one case (2.5%) (Table 6). Hughes and Revington [26] also reported on one case with sensor disorders in the scar out of 75 patients in their study. The access to the proximal medial tibia is relatively safe because there are no nerve stumps above the bone surface [44].

In our study, a dip in bone was found in one patient (2.5%), a 24-year-old woman, 6 months postoperatively. This complication was not a problem for the patient because the dip was noticeable only to palpation and did not affect the aesthetics of the leg. This kind of complication was also found by Hughes and Revington [26] in their study.

A fracture of the proximal tibia is a rarely described complication of the cancellous bone harvesting procedure and was not found in our own data. There have been case reports of tibia fracture after bone transplantation [28]. Hughes and Revington [26] found this complication in two of the 75 analyzed cases. These authors emphasize, however, that the real incidence of this complication is difficult to assess. The occurrence of fracture sometime after surgery does not clearly indicate that this is a consequence of the treatment. Second, some cases of proximal tibia fractures remain unnoticed by the patient and heal spontaneously. In a study of the mechanical strength of the tibia after cancellous bone harvesting carried out on cadavers, it was found that even making a hole in the cortical layer of the tibia 10 mm in diameter does not affect the substantial strength of the bones [37]. There have been reports on the risk of iliac crest fractures during the surgery, as well as in the postoperative run [15,45]. Fasolis et al. [46] in the group of 61 patients found one fracture of the iliac crest during surgery and one in the first postoperative day.

In the analyzed data a wound infection occurred in one patient, a 61-year-old man. The same patient developed increased postoperative bleeding. It is possible that the bleeding led to the partial relaxation of sutures. In addition, the need to replace the compression dressing could cause additional wound infection. This resulted in a need for removing two stitches 4 days postoperatively to allow the evacuation of the pus and the extension of the antibiotic therapy to 14 days. The consequence of this was the prolongation of the wound healing to 5 weeks postoperatively and the formation of an unattractive scar in the lower limb. It should be noted that this complication has also been found only quite rarely by other authors. Hughes and Revington [26] observed it in only one of 75 cases.

### Limitations of the Study

In our studies, the survival of the bone graft was only assessed on the basis of OPGs. It was assumed that the lack of any pathological changes in the bone structure of the graft and around the augmented area was proof of the proper healing of the grafted bone. However, the visual assessment of the bone based on the radiological examination seems to be a limitation of the performed studies. In the future, the authors plan to undertake studies in which bone samples from the area augmented with the bone graft from the tibia will be collected for histopathological examinations. It seems to be possible to perform this without any bioethical considerations during the preparation of the site for the dental implant placement. Moreover, in the process of qualifying the patients for cancellous bone harvesting from the proximal tibia only the data from anamnesis and the results of P-A and lateral X-rays of the knee joints were taken into account. No additional medical examination was performed to identify any pre-existing conditions. With regard to self-ambulation or limping, the patient’s previous surgical history of the lower extremity, even on the contralateral side, and the presence or absence of coexisting orthopedic problems, such as alignment abnormalities or arthritis of the affected lower extremity, can be of fundamental importance.

## 5. Conclusions

(1) The use of the proximal tibia as a donor site enables procurement of large amounts of cancellous bone (15.09 cc in average) and the medial approach to the bone is characterized by a relatively short surgery time (mean 44.12 min), short skin incisions (25.21 mm), and the lack of intraoperative complications.
(2) Harvesting of the cancellous bone from the proximal tibia allows for early patient mobilization after surgery (average 5.4 h after the procedure), although gait disturbances were observed in 72.5% of patients which last for an average of 20.59 days and require the use of walking aids in 25.0% of patients for a mean period of 13.30 days with the use of analgesics for an average of 7.95 days.
(3) The proximal tibia as a donor site for cancellous bone is characterized by a low risk of early and late complications, which include excessive bleeding, wound infection, lengthy healing time, scars, loss of sensation around the scars, aching, a dip in bone, swelling and tenderness.
(4) The ability to obtain large amounts of cancellous bone and a low risk of intra- and postoperative complications make the proximal tibia an attractive donor site for the bone grafting procedures in maxillofacial surgery.

## Figures and Tables

**Table 1:** Descriptive statistics of selected intraoperative parameters obtained during the procedure of cancellous bone harvesting from the proximal tibia in the general group of patients.

	The Whole Group (*n* = 40)		
	Mean ± SD	Min	Max
The length of the skin incision [mm]	25.21 ± 3.23	20	33
Surgery time from the skin incision until the end of the skin suturing [min]	44.21 ± 3.59	37	52
Surgery time from the bone opening until the beginning of the suturing [min]	23.74 ± 3.01	19	29
Soft tissue management time [min]	20.47 ± 2.13	17	25
Quantity of the harvested cancellous bone [cc]	15.09 ± 3.75	8	21

**Table 2:** Descriptive statistics of selected intraoperative parameters obtained during the procedure of cancellous bone harvesting from the proximal tibia in women and men.

	Women (*n* = 26)			Men (*n* = 14)			
	Mean ± SD	Min	Max	Mean ± SD	Min	Max	*p*-Level
The length of the skin incision [mm]	26.10 ± 3.03	21	33	23.36 ± 2.90	20	28	0.007 ^a^*
Surgery time from the skin incision until the end of the skin suturing [min]	44.17 ± 3.91	37	52	44.29 ± 2.95	39	49	0.924 ^a^
Surgery time from the bone opening until the beginning of the suturing [min]	23.93 ± 3.31	19	29	23.36 ± 2.34	20	28	0.564 ^a^
Soft tissue management time [min]	20.24 ± 1.96	18	25	20.93 ± 2.46	17	25	0.378 ^b^
Quantity of the harvested cancellous bone [cc]	15.28 ± 4.12	8	21	14.71 ± 2.92	9	20	0.337 ^b^

* an asterisk indicates a statistically significant result at *p* = level < 0,05. ^a^ as estimated by Student’s *t*-test; ^b^ as estimated by U Mann-Whitney test.

**Table 3:** Descriptive statistics of selected parameters characterizing the early postoperative period after the procedure of cancellous bone harvesting from the proximal tibia in the general group of patients.

	The Whole Group (*n* = 40)		
	Mean ± SD	Min	Max
Time from surgery endpoint until patient mobilization [h]	5.40 ± 1.14	4	11
No of days with pain at the donor site requiring pain killers	7.95 ± 2.55	1	14

**Table 4:** Descriptive statistics characterizing difficulties in ambulation after the procedure of cancellous bone harvesting from the proximal tibia in the general group of patients.

	The Whole Group (*n* = 40)			
	No of Cases (%)	Mean ± SD	Min	Max
No of days with limping	29 (72.5%)	20.59 ± 14.76	2	86
No of days with a walking aid	10 (25.0%)	13.30 ± 4.30	7	21

**Table 5:** The occurrence of early complications after the procedure of cancellous bone harvesting from the proximal tibia.

Problem	No of Cases
Excessive bleeding	3 (7.5%)
Wound infection	1 (2.5%)

**Table 6:** The occurrence of late complications after the procedure of cancellous bone harvesting from the proximal tibia.

Problem	No of Cases
Lengthy healing time	1 (2.5%)
Scar	5 (12.5%)
Loss of sensation around the scar	1 (2.5%)
Aching	2 (5.0%)
Dip in bone	1 (2.5%)
Swelling and tenderness	1 (2.5%)

## Data Availability

The data presented in this study are available on request from the corresponding authors. Publicly data sharing is not applicable to this article due to privacy policy.
